# Quantitative and qualitative characteristics of cell wall components and prenyl lipids in the leaves of *Tilia x euchlora* trees growing under salt stress

**DOI:** 10.1371/journal.pone.0172682

**Published:** 2017-02-24

**Authors:** Anna Milewska-Hendel, Aneta H. Baczewska, Katarzyna Sala, Wojciech Dmuchowski, Paulina Brągoszewska, Dariusz Gozdowski, Adam Jozwiak, Tadeusz Chojnacki, Ewa Swiezewska, Ewa Kurczynska

**Affiliations:** 1 Department of Cell Biology, Faculty of Biology and Environmental Protection, University of Silesia, Katowice, Poland; 2 Polish Academy of Sciences Botanical Garden–Center for the Conservation of Biological Diversity, Warsaw, Poland; 3 Warsaw University of Life Sciences–SGGW, Warsaw, Poland; 4 Institute of Environmental Protection–National Research Institute, Warsaw, Poland; 5 Institute of Biochemistry and Biophysics–Polish Academy of Sciences, Warsaw, Poland; University of Massachusetts Amherst, UNITED STATES

## Abstract

The study was focused on assessing the presence of arabinogalactan proteins (AGPs) and pectins within the cell walls as well as prenyl lipids, sodium and chlorine content in leaves of *Tilia x euchlora* trees. The leaves that were analyzed were collected from trees with and without signs of damage that were all growing in the same salt stress conditions. The reason for undertaking these investigations was the observations over many years that indicated that there are trees that present a healthy appearance and trees that have visible symptoms of decay in the same habitat. Leaf samples were collected from trees growing in the median strip between roadways that have been intensively salted during the winter season for many years. The sodium content was determined using atomic spectrophotometry, chloride using potentiometric titration and poly-isoprenoids using HPLC/UV. AGPs and pectins were determined using immunohistochemistry methods. The immunohistochemical analysis showed that rhamnogalacturonans I (RG-I) and homogalacturonans were differentially distributed in leaves from healthy trees in contrast to leaves from injured trees. In the case of AGPs, the most visible difference was the presence of the JIM16 epitope. Chemical analyses of sodium and chloride showed that in the leaves from injured trees, the level of these ions was higher than in the leaves from healthy trees. Based on chromatographic analysis, four poly-isoprenoid alcohols were identified in the leaves of *T*. *x euchlora*. The levels of these lipids were higher in the leaves from healthy trees. The results suggest that the differences that were detected in the apoplast and symplasm may be part of the defensive strategy of *T*. *x euchlora* trees to salt stress, which rely on changes in the chemical composition of the cell wall with respect to the pectic and AGP epitopes and an increased synthesis of prenyl lipids.

## Introduction

Salt stress is a complex process that includes changes in plants on the physiological, histological, cellular and molecular levels by limiting nutrient uptake and disrupting the ionic balance [1–5].

The cell wall is an integral part of a plant cell, it is metabolically active and dynamically changes in response to internal and external factors. The diversity of the cell wall chemical components is an expression of the changes in cells that are under the influence of various factors. Pectins are components that are subjected to changes in relation to the operating biotic and abiotic factors [6–8]. Quantitative and qualitative changes in the pectin composition have been described in plants growing under different abiotic stresses [9–15] including salinity [16,17]. Arabinogalactan proteins (AGPs) play an important role in the control of plant development [18–22], and changes in the AGPs indicate that they respond to both biotic and abiotic factors including salinity [23–28].

It is postulated that poly-isoprenoids may play a role in the adaptation of trees to abiotic stressors [29,30]. One possible mechanism of this involvement might comprise two nonexclusive processes, i.e. (i) an increase in the fluidity of cellular membranes (to support the cellular demand for the enhanced transport of lipids and proteins that have been newly synthesized in the cell in response to stress) and (ii) protection (via a suicide mechanism) of the cellular components against the reactive oxygen species (ROS) that are massively generated in response to stress. Poly-isoprenoids belong to the group of prenyl lipids primarily detected in the cell membrane [31], and the postulated biological role of prenols is their impact on the fluidity and stability of the cell membranes [32,33]. Presumably, they also act as "scavengers" of free radicals [30,34,35]. There is very little information describing the role of prenyls in the plant response to stress conditions [36]. Analyses that have been performed so far suggest that the high content of prenols in the leaves of *T*. *x euchlora* trees may be an adaptation to growth in salt stress because the prenol content changes with the health status of trees [30].

The soil contamination that is caused by the NaCl that is used to de-ice slippery roads in winter is now recognized as one of the major causes of the nutrient disorders and death of urban trees (e.g., [37–46]). Research that was carried out in the center of Warsaw by Dmuchowski et al. [47] showed that over a period of 34 years, more than half (59%) of sidewalk trees had died. The greatest losses were found for *T*. *x euchlora* (62%). Moreover, long term studies performed in that area, which is a typical example of the influence of salinity on trees from the urban ecosystems, showed that within the tree population, some trees have signs of damage and some do not. Such observations forced us to analyze the status of “health” and “injured” trees on the histological and cellular level with respect to composition of the cell wall and the of prenol content in the leaves.

The brief literature review presented above indicates that there may be a relationship between prenyl lipids, sodium and chlorine level, the chemical composition of cell wall and the health status of trees. Thus, the study was focused on the above-mentioned parameters as characterized in leaves from *T*. *x euchlora* trees with and without signs of damage that were all growing in the same salt stress conditions. The reason for undertaking these investigations was observations that had been carried out for many years, which indicated that there are trees that present a healthy appearance and trees that have visible symptoms of decay in the same habitat. Thus, the aim of the present study was to attempt to answer the question of whether healthy trees can develop a defense strategy based on changes in 1) the cellular distribution of selected pectic and AGP epitopes in their leaves, and 2) the content of sodium, chloride and prenyl lipids in order to overcome the harmful effects of salt stress.

## Materials and methods

No specific permissions were required for the study area. We obtained permissions to collect samples from the waste of the Department of Urban Greenery of the Municipal Office. The field studies did not involve endangered or protected species. Specific location of study: 52°12'15", 20°59'13"E.

### Plant material

The study was performed on Crimean linden (*Tilia x euchlora*) trees, which is a sterile hybrid of small-leaved lime (*Tilia cordata* Mill.), and *Tilia dasystyla* Steven trees, which is a tree that has been known since the mid-nineteenth century [48]. The leaves were collected from the external belt of the crown around its entire circumference at heights of 2 m to 4 m (twigs were cut off with pruning shears). In 2012, samples were collected from 80-year-old trees growing in the median strip between roadways that are characterized by high intensity traffic, which have been intensively salted during the winter season for many years. Assessment of tree conditions had been done on two dates in mid-July and mid-September 2011 (and many previous years), and were based on observing any adverse changes in leaves (e.g., browning edges of the leaves, chlorosis, and necrosis) [49]. While collecting the samples in mid-July, the injuries of the leaves had just begun to appear. The injuries fully developed in September. Therefore, the health categories “healthy” and “injured” were determined based on the observations that had been made in September of the previous year. The health condition of the trees was confirmed by the observations that were made in September of the year of samples collection. In order to limit the risk of errors, we selected only trees that were characterized by the most often repeated health status and those that had a consistent content of chloride and sodium since 1996 (our own studies). “Healthy” trees were those with minor injuries to the leaf blade (less than 5% of the leaf blade area) and “injured” trees were characterized by a high degree of leaf blade damage (over 50% of the leaf blade area).

### Chemical analysis of leaves

Leaf samples for the chemical analysis were collected separately from 16 trees (8 healthy and 8 injured) in mid-July 2012. The leaves were separated from the twigs, placed in linen bags and then brought to the laboratory and dried at 70°C. The dried material was ground to a powder using an impactor mill. To determine the sodium content, the powdered samples were dry-mineralized in a muffle oven [50] and analyzed by atomic spectrophotometry using a Perkin Elmer 1100B spectrophotometer [51]. Chloride was determined by potentiometric titration using an ion-selective electrode and an Orion Star Plus ion meter [52]. To provide quality control (QC), the elemental content in the plant samples was determined using certified reference materials, including apple leaves from the NIST–USA and beech leaves from Sigma-Aldrich.

The obtained results were in good agreement with the certified values. The recovery range was 94.6% for chloride content and 104.5% for sodium content.

To determine poly-isoprenoid, parallel samples of dry leaf powder that were obtained in the same manner as above (50 mg) were subjected to extraction with a mixture of acetone:hexane (1:1, v/v), after which the extracts were supplemented with an internal standard (50 μg of Prenol-15, C = 1 μg/μl). Lipids were extracted at 37°C for 30 min. The extract was removed by centrifugation and decantation. The tissue was re-extracted four times with new portions of the solvent mixture. All extracts were pooled and evaporated under a stream of nitrogen. The dry residue was dissolved in 0.5 ml of hydrolyzing mixture (7.5% KOH in a mixture of water/toluene/ethanol (1:6.6:5.5 v/v/v) containing 0.2% pyrogallol) and incubated at 95°C for 1 h. When the sample reached room temperature, 1 ml of water, 1 ml of hexane and 0.5 ml of saline were added and mixed vigorously. After phase separation, the organic layer was removed, and then the water phase was re-extracted with 1 ml of hexane three additional times. The combined organic phases were evaporated under a stream of nitrogen. Lipids were purified as described earlier by Skorupinska-Tudek et al. [53]. Briefly, quantitative and qualitative analyses of the poly-isoprenoids were performed using a HPLC/UV apparatus (Waters) equipped with a Waters UV detector (Waters 2487). Separation was performed using reversed-phase column ZORBAX XDB-C18 (4.6 x 75 mm, 3.5 μm) (Agilent, USA). We used a combination of linear gradient mixtures of solvent A (90% methanol in water, v/v) and solvent B (50% methanol, 25% hexane and 25% isopropanol v/v/v) at a flow rate of 1.5 ml/min. The analysis time was 25 minutes and the injection volume was 10 μl. For other details, see Jozwiak et al. [54]. Poly-isoprenoid compounds were identified by comparing their retention times and absorption spectra with the corresponding parameters of standard substances (using an external standard—Pren-9, Pren-11 to Pren-23, and Pren-25 from the Collection of Polyprenols, IBB PAS). Chromatograms were integrated using the Empower Pro program (Waters). The content of the identified compounds was expressed as mg/g dry weight of the plant tissue. Each analysis was performed in triplicate the presented data are the mean ± SD of three independent analyses.

### Immunohistochemistry

For the histological study, samples were collected from eight trees that were classified as healthy and eight trees that were classified as injured. Branches with leaves were collected from the southern (sunlit) and northern (shady) side of the trees. After cutting, the branches were inserted into water and taken to the laboratory for further procedures. The plant material was divided into four variants: 1) leaves from the southern side of healthy trees; 2) leaves from the southern side of injured trees; 3) leaves from the northern side of injured trees and 4) leaves from the northern side of healthy trees. Samples were fixed in 4% paraformaldehyde and 2% glutaraldehyde in PBS (Phosphate Buffered Saline) and were then dehydrated and embedded in Steedman’s wax according to the procedures described previously [55,56]. Samples were cut into 7 μm cross-sections using a rotary microtome (Zeiss Hyrax M40) and were then attached to microscope slides coated with Mayer’s egg albumen, dewaxed in alcohol and rehydrated through a degraded ethanol series. For immunohistochemical analysis, samples were blocked with 2% bovine serum albumin (BSA) and 2% fetal calf serum (FCS) in PBS for 30 min. Then, specimens were incubated with the primary monoclonal antibodies for pectin detection, i.e., LM5, LM6, LM19, LM20; and AGP detection, i.e., JIM8, JIM13, JIM16, and LM2 (Table 1; diluted 1:20 in a blocking buffer for 1.5 h at RT). Subsequently, slides were incubated for 1.5 h with secondary antibodies labelled with AlexaFluor488, then diluted 1:100 in a blocking buffer. Details for these procedures have previously been described [55,56]. As a control, sections were incubated with a blocking buffer instead of primary antibodies and with AlexaFluor488-labelled secondary antibodies. Observations and documentation were carried out using a Nikon Eclipse Ni-U microscope equipped with a Nikon Digital DS-Fi1-U3 camera with corresponding software (Nikon, Tokyo, Japan) using a maximum excitation wavelength of 490 nm and obtaining maximum emission at 590 nm. Immunohistochemical analyses were performed on at least three samples (several transversal sections from each leaf, separately for the leaf lamina and petiole) from different trees. The photos are representative of the obtained results.

**Table 1:** List of the monoclonal antibodies used in the current study, the epitopes they recognized and references.

Antibody	Epitope	References
*Pectins*		
LM19	Low methyl-esterified HG	Verhertbruggen et al. 2009
LM20	High metyl-esterified HG	Verhertbruggen et al. 2009
LM5	(1→4)-ß-D-galactan	Jones et al. 1997
LM6	(1–5)-α-L-arabinan	Willats et al. 1998
*AGPs*		
JIM8	Arabinogalactan	Pennell et al. 1991, Yates et al. 1996
JIM13	AGP glycan	Knox et al. 1991, Yates et al. 1996
JIM16	AGP glycan	Knox et al. 1991, Yates et al. 1996
LM2	ß-linked GlcA	Smallwood et al. 1996, Yates et al. 1996

### Statistical analysis

The obtained results were statistically analyzed in Statistica 10 using a one-way analysis of variance. Multiple comparison procedures were performed using Tukey’s test to compare the means of the chemical composition of the leaves. Based on the analysis, the groups of homogeneous means were distinguished. Immunohistochemical data were also subjected to statistical analysis using the Kruskal–Wallis test to compare the means for all of the AGP and pectic epitopes (Tables 2 and 3). For all of the analyses, the significance level was set at 0.05.

**Table 2:** Means of the AGP (± SE) and pectic epitopes in the petiole and their comparisons based on the Kruskal-Wallis test.

	shady-healthy	sun-healthy	shady-injured	sun-injured	*P-value*
AGP all–petiole	1.44a ± 0.18	1.29a ± 0.18	1.64a ± 0.18	1.58a ± 0.19	*0*.*351*
Pectins all–petiole	2.08b ± 0.22	1.44b ± 0.18	2.04b ± 0.20	0.80a ± 0.15	*<0*.*001*
Petiole JIM8	0.90a ± 0.32	0.70a ± 0.26	1.30a ± 0.32	1.15a ± 0.36	*0*.*534*
Petiole JIM13	2.50a ± 0.31	2.55a ± 0.34	2.45a ± 0.34	2.50a ± 0.34	*0*.*993*
Petiole JIM16	0.20a ± 0.09	0.00a ± 0.00	0.70a ± 0.28	0.40a ± 0.11	*0*.*028*
Petiole LM2	2.15a ± 0.40	1.90a ± 0.36	2.10a ± 0.35	2.25a ± 0.41	*0*.*804*
Petiole LM5	0.55a ± 0.22	1.50b ± 0.25	0.45a ± 0.11	0.60ab ± 0.18	*0*.*004*
Petiole LM6	2.55a ± 0.42	2.80a ± 0.48	1.90a ± 0.35	2.35a ± 0.40	*0*.*343*
Petiole LM19	1.60b ± 0.22	1.25b ± 0.19	3.00b ± 0.40	0.00a ± 0.00	*<0*.*001*
Petiole LM20	3.60b ± 0.48	0.20a ± 0.09	2.80b ± 0.42	0.25a ± 0.10	*<0*.*001*

**Note:** Different letters indicate statistically significant differences. The scale is from 0 to 5, where the individual values represent 5 (++) very strong signal, 4 (+) strong signal, 3 (++/-) epitope present in the most of cells, 2 (+/-) epitope present in the part of cells, 1 (+/--) epitope present in the individual cells; 0 (-) epitope not detected.

**Table 3:** Mean levels (± SE) of the AGP and pectic epitopes in the leaf lamina and their comparisons based on the Kruskal-Wallis test.

	shady-healthy	sun-healthy	shady-injured	sun-injured	*P-value*
AGP all–leaf lamina	1.79b ± 0.13	1.79b ± 0.11	1.51ab ± 0.12	1.27a ± 0.12	*0*.*009*
PECTINS all–leaf lamina	2.46b ± 0.15	1.59a ± 0.13	2.98b ± 0.17	1.62a ± 0.16	*<0*.*001*
Leaf lamina JIM8	0.86a ± 0.15	1.49a ± 0.24	1.23a ± 0.21	1.14a ± 0.14	*0*.*299*
Leaf lamina JIM13	1.94a ± 0.28	2.06a ± 0.24	2.60a ± 0.21	2.66a ± 0.17	*0*.*168*
Leaf lamina JIM16	1.34b ± 0.17	1.49b ± 0.21	0.00a ± 0.00	0.00a ± 0.00	*<0*.*001*
Leaf lamina LM2	3.03b ± 0.23	2.11a ± 0.22	2.23ab ± 0.21	1.29a ± 0.30	*<0*.*001*
Leaf lamina LM5	0.57a ± 0.12	0.63a ± 0.12	1.23a ± 0.19	0.86a ± 0.13	*0*.*038*
Leaf lamina LM6	2.57ab ± 0.31	2.23a ± 0.27	3.34bc ± 0.37	3.49c ± 0.38	*<0*.*001*
Leaf lamina LM19	3.29b ± 0.24	2.63b ± 0.27	4.14c ± 0.30	0.00a ± 0.00	*<0*.*001*
Leaf lamina LM20	3.43c ± 0.24	0.86a ± 0.15	3.20c ± 0.24	2.14b ± 0.23	*<0*.*001*

**Note:** Different letters indicate statistically significant differences. The scale of 0 to 5, where the individual values represent 5 (++) very strong signal, 4 (+) strong signal, 3 (++/-) epitope present in the most of cells, 2 (+/-) epitope present in the part of cells, 1 (+/--) epitope present in the individual cells; 0 (-) epitope not detected.

## Results

### Chemical composition of the leaves

#### Chlorine and sodium

The content of chloride and sodium in leaves of the healthy trees was significantly lower than in the leaves of injured trees (Table 4; in the remaining text, for simplicity, in the results description “healthy leaves” and “injured leaves” means leaves from trees that were determined to be healthy and injured, respectively). These differences were statistically significant. There were no statistically significant differences between the chloride and sodium content in the leaves collected from the sunlit and shady sides of the crown (Table 4).

**Table 4:** Comparison of the average concentrations of chlorine and sodium in leaves under different health and light conditions.

Status of health trees	Light conditions	Cl [%] Mean with SD	Na [mg/kg] Mean with SD
healthy	shady	0.88a ± 0.11	185a ± 42
	sunlit	0.85a ± 0.10	173a ± 37
injured	shady	1.43b ± 0.09	952b ± 500
	sunlit	1.39b ± 0.07	939b ± 393

**Note:** Groups of means were separated based on the Tukey’s analysis; different letters indicate statistically significant differences.

#### Prenyl lipids

**Fig 1:**
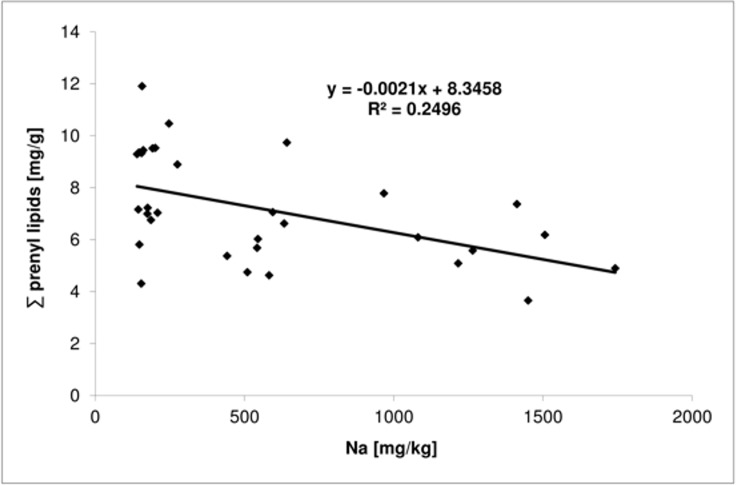
The relation between the sodium content and prenyl lipids in the leaves of Crimean linden.

**Fig 2:**
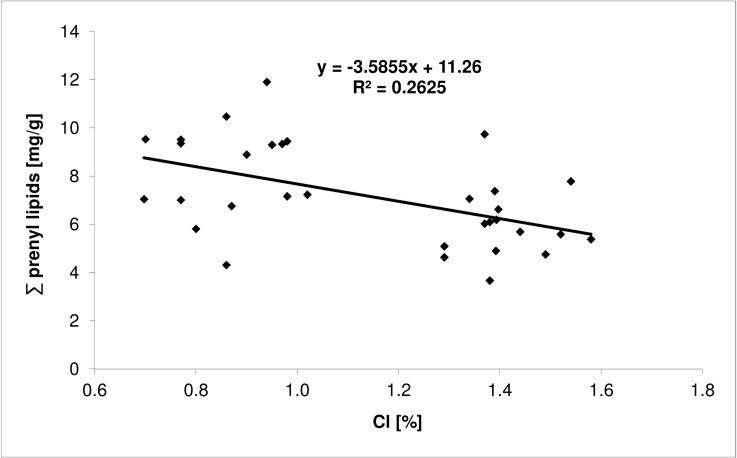
The relation between the chlorine content and prenyl lipids in the leaves of Crimean linden.

Based on chromatographic analysis (HPLC/UV), four prenyl lipids were identified in the leaves of Crimean linden, i.e., Pren-9, -10, -11 and -12. Different light conditions affected only the total content of prenyl lipids (Table 5) but not the profile. Leaves from the sunlit side of the trees had a significantly higher content of these compounds than leaves from the shady side. Moreover, it was found that the total prenyl lipid content was higher in healthy leaves than in injured ones. A strong positive correlation was found between the contents of chloride and sodium and the total content of prenyl lipids (Figs 1 and 2, respectively), and of individual prenyl lipids in the leaves, with the exception of Pren-12. Based on linear regression analysis (Figs 1 and 2), it was found that initially (for lower concentration of chlorine and sodium salt) the prenyl lipid content decreased with increasing chloride and sodium concentrations in the leaves.

**Table 5:** Comparison of the average concentrations of prenyl lipids in leaves under different health and light conditions.

Status of trees health	Light conditions	Prenyl lipids (mg/g of dry leaf weight) Mean with SD				
		Pren-9	Pren-10	Pren-11	Pren-12	∑ prenyl lipids
healthy	shady	0.74 bc ± 0.19	3.52b ± 0.95	2.81b ± 0.74	0.17ab ± 0.03	7.24b ± 1.54
	sunlit	0.80 c ± 0.16	4.71c ± 1.00	3.65c ± 0.80	0.26 c ± 0.03	9.41c ± 1.71
injured	shady	0.49 a ± 0.10	2.50a ± 0.41	1.94a ± 0.35	0.15 a ± 0.05	5.09a ± 0.76
	sunlit	0.65 b ± 0.10	3.48b ± 0.78	2.67b ± 0.62	0.20 b ± 0.02	6.99b ± 1.40

**Note:** Groups of means were separated based on the Tukey’s analysis; different letters indicate statistically significant differences.

### Immunohistological analysis of pectic and AGP epitopes

#### AGP epitopes, leaf lamina, sunlit side (Table 6)

**Fig 3:**
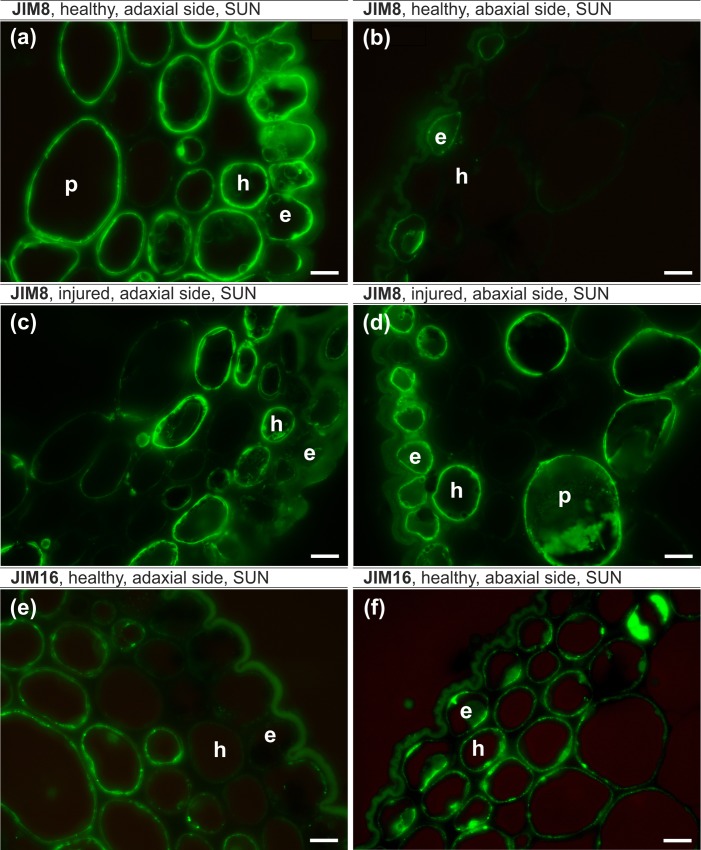
**JIM8 epitope (a-d) and JIM16 epitope (e-f) leaf lamina, sunlit side.** (a,b,e,f) – healthy leaf, (c,d) – injured leaf, (a,c,e) – adaxial side, (b,d,f) – abaxial side. e – epidermal cells, h – hypodermal cells, p – parenchyma. Bars, 10 μm.

**Fig 4:**
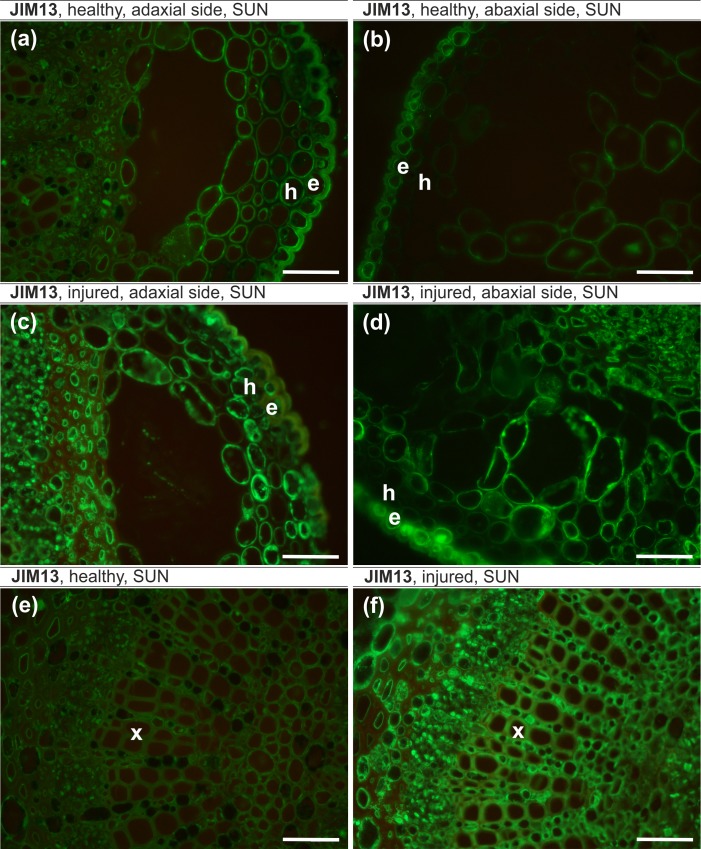
JIM13 epitope, sunlit side. (a,b,e) – healthy leaves, (c,d,f) – injured leaves, (a,c) – adaxial side, (b, d) – abaxial side. e – epidermal cells, h – hypodermal cells, x – xylem cells. Bars, 50 μm.

**Fig 5:**
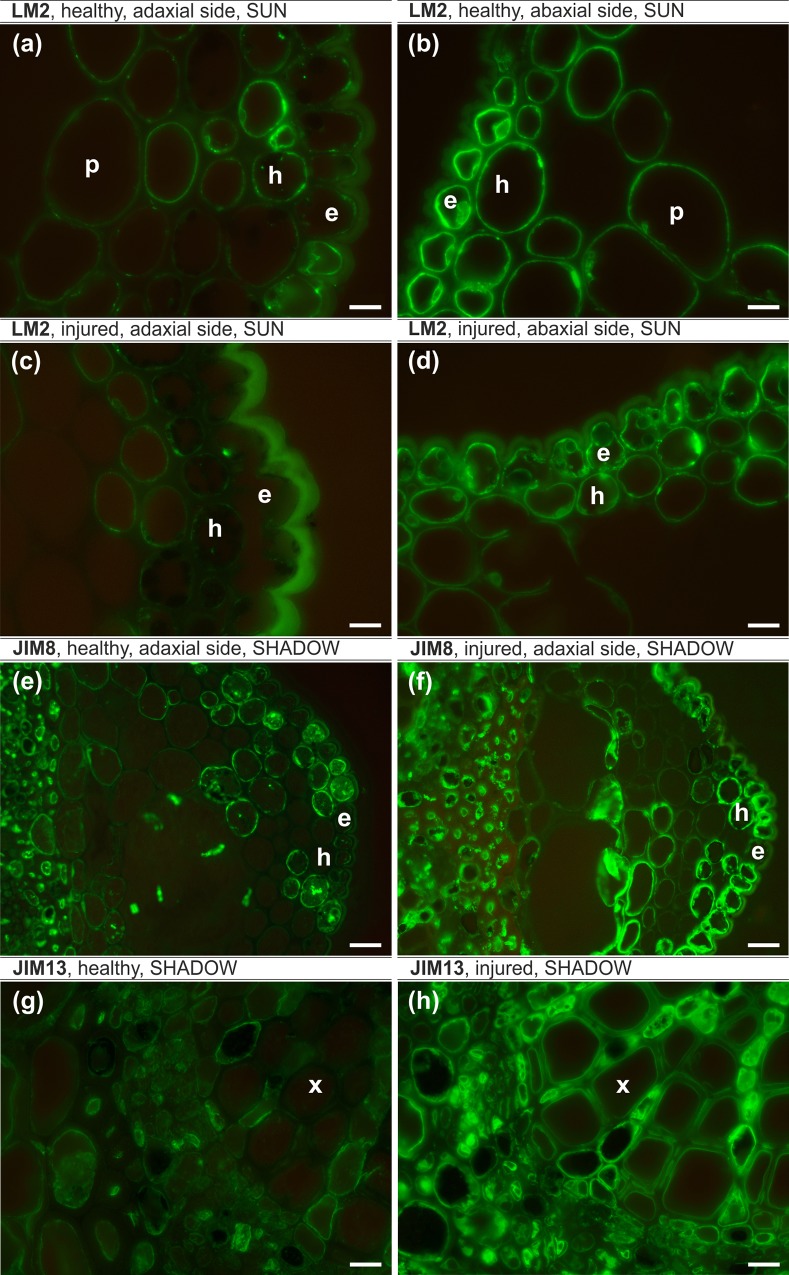
**LM2 epitope, leaf lamina, sunlit side (a-d), JIM8 epitope, leaf lamina, shady side (e,f), JIM13 epitope, leaf lamina, shady side (g-h)** (a,b,e,g) – healthy leaves, (c,d,f,h) – injured leaves (a,c,e,f) – adaxial side, (b,d) – abaxial side. e – epidermal cells, h – hypodermal cells, x – xylem cells. Bars, 10 μm.

The epitope recognized by JIM8 antibodies in healthy leaves were localized in the cytoplasm, cell wall and plasmalemma in the majority of the adaxial epidermal cells and were abundant in parenchyma and hypodermis cells (Fig 3A). However, on the abaxial side, JIM8 was only observed in the individual epidermal cells (Fig 3B). In injured leaves, JIM8 antibodies were present in the cytoplasm in the individual cells of all of the analyzed tissues independent of the leaf side (Fig 3C and 3D). The JIM16 epitope in healthy leaves was present in the cytoplasm and outer periclinal walls in some abaxial epidermal cells, in the cell walls of most abaxial hypodermal cells and in the cytoplasm and plasmalemma of abaxial and adaxial parenchyma cells (Fig 3E and 3F). JIM16 was not detected in any cells of injured leaves. JIM13 antibodies were present in the cytoplasm and plasmalemma or cell walls in healthy (apart from the abaxial hypodermis) and injured leaves (Fig 4A–4D). The presence of the JIM13 epitope in the xylem was detected only in injured leaves (Fig 4E and 4F). The LM2 epitope was present in the epidermis and hypodermis on the abaxial side and in the parenchyma cells on the abaxial and adaxial sides of injured leaves (Fig 5C and 5D) and on both sides in all of the analyzed tissues of healthy leaves (Fig 5A and 5B).

**Table 6:** Analysis of the distribution of the JIM8, JIM16, JIM13, and LM2 epitopes associated with the AGPs in various tissues.

AGP		LEAF LAMINA				PETIOLE					LEAF LAMINA				PETIOLE			
		sunlit		shady		sunlit		shady			sunlit		shady		sunlit		shady	
		healthy	injured	injured	healthy	healthy	injured	injured	healthy		healthy	injured	injured	healthy	healthy	injured	injured	healthy
**JIM8**	Adaxial epidermis	++/- c, p, w	+/--c, p	++/- c, p, w	+/- c, p	++/- c, p	++/- c, p	++/- c, p	++/- c, p	**JIM13**	++/- w	+/- c, p	++/- c, p	+ c, p	+ c, w	+ c, w	+ c, p	+ c, p
	Abaxial epidermis	+/--c, p	++/- c, p	+/--c, w	+/--c, w						+ w	+ c, p	++/- c, p	+ c, p				
	Adaxial hypodermis	++/- c, p	+/--c	++/- c, p, w	+/- p	-	-	-	-		++/- w	++/- c, w	++/- c, p	++/- c, p	+/--c	+/--c	+/--c	+/--c
	Abaxial hypodermis	-	+/--c	-	-						-	+/--p	-	-				
	Adaxial parenchyma	++/- c, p	+/--c	+/- c, p, w	+/--c, p	-	-	-	-		+/- w	++/- c, w	++/- c, p	+/--p	+/--w	+/--w	+/--w	+/--w
	Abaxial parenchyma	-	+/--c	-	-						+/- p	+/- c, p	+/- c, p	+/--p				
	Xylem	-	-	-	-	-	+/- l	+/- w, l	-		-	+ l	+ l	-	+ w, l	+ w, l	+/--w, l	+/--l
**JIM16**	Adaxial epidermis	-	-	-	++/- c, p	-	-	-	-	**LM2**	+/--w	-	+/- w	+ c, w	+ c, w	+ c, w	+ c, w	+ c, w
	Abaxial epidermis	+/- c, w	-	-	-						+ c, w	+ c, w	+ c, w	+ c, w				
	Adaxial hypodermis	-	-	-	+/- c, p	-	+/--c, p	++/- c, p	+/--c, p		+/- w	-	+/- w	+ w	++/- c, w	+ c, p	++/- w	+ p
	Abaxial hypodermis	++/- w	-	-	+/- p						++/- w	+ w	++/- w	++/- w				
	Adaxial parenchyma	+/- c, p	-	-	+/- c, p	-	+/--c, p	-	-		+/- w	+/--w	++/- w	+ w	+/--w	+/--w	+/--w	+/--w
	Abaxial parenchyma	+/- c, p	-	-	+/--w, p						++/- w	+/--w	++/- w	++/- w				
	Xylem	-	-	-	-	-	-	-	-		-	-	-	-	-	-	-	-

c – cytoplasm, l – middle lamella, p – plasma membrane, w – cell wall; ++ very strong signal, + strong signal, ++/- epitope present in the most of cells, +/- epitope present in the part of cells, +/--epitope present in the individual cells;—epitope not detected.

#### AGP epitopes, leaf lamina, shady side (Table 6)

The JIM8 epitope was more abundant on the adaxial side of the leaves, and the localization of signals in the cell was significantly different in injured leaves compared to healthy ones (Fig 5E and 5F). The JIM16 epitope was present only in plasmalemma adjacent to the outer and inner periclinal epidermal walls on the adaxial side and on both sides of the leaf lamina in the hypodermis and parenchyma in healthy leaves. The JIM13 epitope was found in both healthy and injured leaves but was more abundant in injured leaves, especially in the vascular tissue (Fig 5G and 5H). The LM2 antibody was present in healthy and injured leaves with no significant differences.

Summarizing, statistically significant differences comprising the AGP epitopes in all of the variants were present in the case of both the JIM16 and LM2 epitopes (Table 3).

#### AGP epitopes, petiole, sunlit side (Table 6)

The JIM8 epitope was detected only in the cytoplasm and plasmalemma of epidermal cells in both of the analyzed variants. The JIM16 epitope was not present in healthy leaves at all but a weak JIM16 signal was observed in the hypodermal and parenchyma cells in injured leaves. There were no significant differences in the case of the JIM13 and LM2 epitopes in either of the analyzed variants.

#### AGP epitopes, petiole, shady side (Table 6)

**Fig 6:**
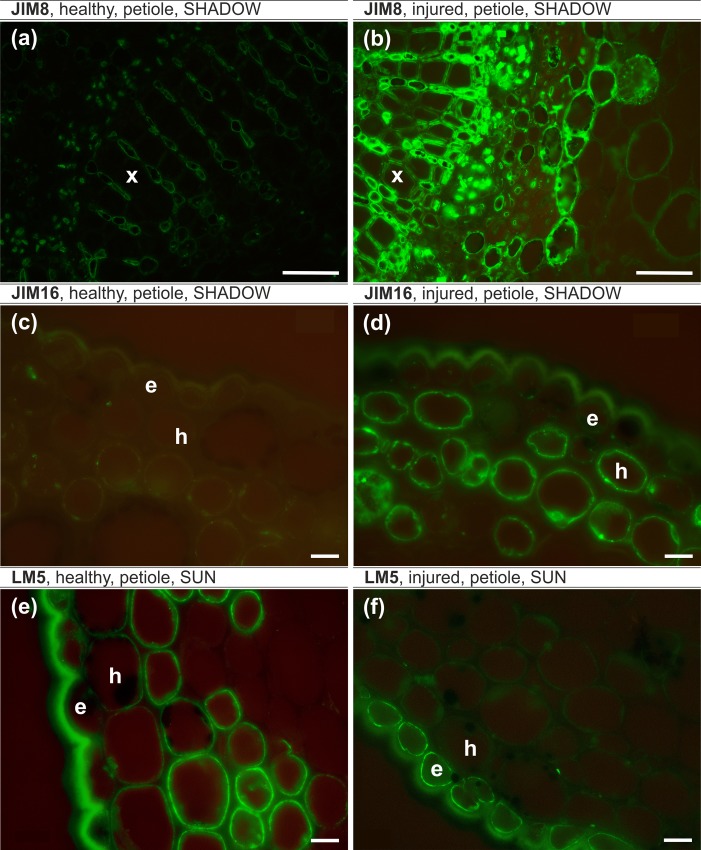
**JIM8 epitope, petiole, shady side (a,b), JIM16 epitope, petiole, shady side (c,d), LM5 epitope, petiole, sunlit side (e,f).** (a,c,e) – healthy leaf, (b,d,f) – injured leaf. e – epidermal cells, h – hypodermal cells, x – xylem cells. Bars (a,b), 50 μm; (c-f), 10 μm.

The occurrence of the JIM8 epitope was different in the xylem tissue in that it was not observed in healthy leaves (Fig 6A and 6B). The JIM16 epitope was detected only in the hypodermis in healthy and injured leaves, but the fluorescence signal was more abundant in injured leaves (Fig 6C and 6D). There were no significant differences in the case of the JIM13 and LM2 epitopes in either of the analyzed variants.

No statistically significant differences were detected regarding the presence of AGPs in the petiole.

#### Pectic epitopes, leaf lamina, sunlit side (Table 7)

**Fig 7:**
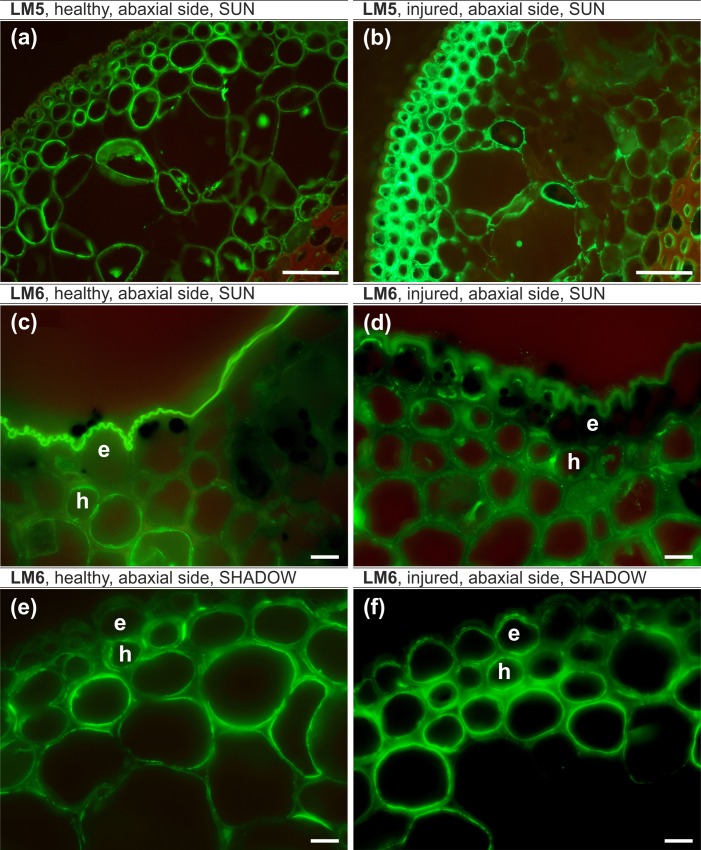
**LM5 epitope, leaf lamina, sunlit side (a,b), LM6 epitope, leaf lamina, sunlit side (c,d) LM6 epitope, leaf lamina, shady side (e,f).** (a,c,e) – healthy leaf, (b,d,f) – injured leaf, (a-f) – abaxial side. e – epidermal cells, h – hypodermal cells. Bars, 10 μm.

**Fig 8:**
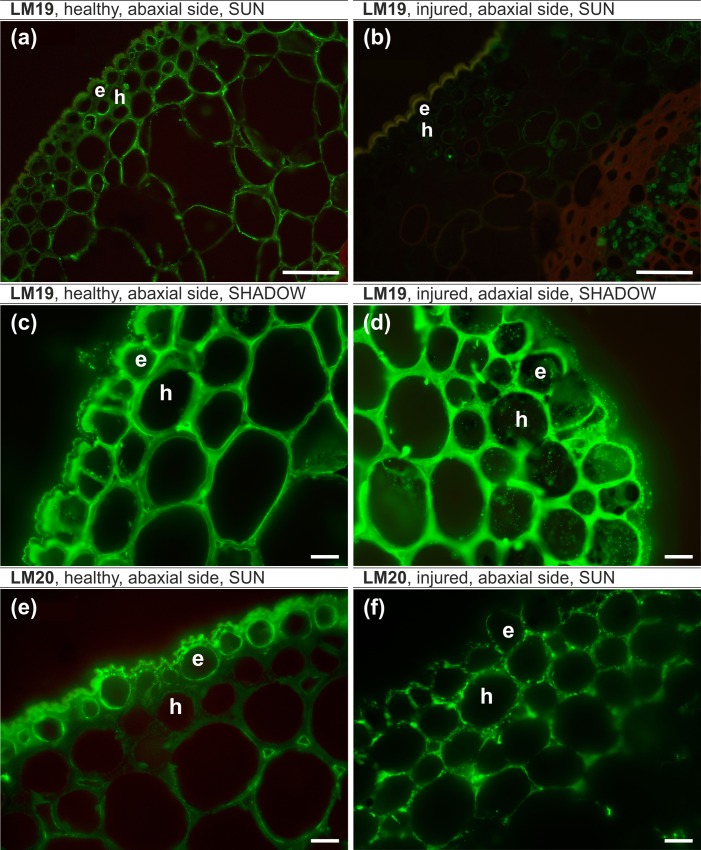
**LM19 epitope, leaf lamina, sunlit side (a,b) LM19 epitope, leaf lamina, shady side (c,d) LM20 epitope, leaf lamina, sunlit side (e,f).** (a,c,e) – healthy leaf, (b,d,f) – injured leaf, (a,b,c,e,f) – abaxial side, (d) – adaxial side. e – epidermal cells, h – hypodermal cells. Bars (a,b), 50 μm, (c-f), 10 μm.

The LM5 epitope occurred in all of the analyzed leaves and was more abundant in injured leaves. In healthy leaves, it was present in intercellular spaces (Fig 7A and 7B). The LM6 epitope was present in all of the analyzed tissues of both healthy and injured leaves but the signal intensity was stronger in injured leaves (Fig 7C and 7D). The LM19 epitope was present only in healthy leaves (Fig 8A and 8B). The LM20 epitope was present in the abaxial epidermis and in the intercellular spaces of the hypodermis and parenchyma in healthy leaves (Fig 8E). In injured leaves, this epitope was present in most of the cell walls of the analyzed tissues (Fig 8F).

**Table 7:** Analysis of the distribution of the LM5, LM6, LM19 and LM20 epitopes associated with pectins in various tissues.

PECTINS		LEAF LAMINA				PETIOLE					LEAF LAMINA				PETIOLE			
		Sunlit		shady		sunlit		shady			sunlit		shady		sunlit		shady	
		healthy	injured	injured	healthy	healthy	injured	injured	healthy		healthy	injured	injured	healthy	healthy	injured	injured	healthy
**LM5**	Adaxial epidermis	-	-	-	-	+/- w	+/- w, c	+/--w	-	**LM19**	+ w	-	++ w, p, c	+ w	+/- w	-	+ w	+/- w
	Abaxial epidermis	+/--w, l	+/--w	-	-						+ w	-	++ w	+ w				
	Adaxial hypodermis	+/- w, l	+/- w	+/- w	+/- w	++/- w	-	+/--w	+/- w		+ w	-	++ w, p, c	+ w, l	+/- w	-	+ w	+/- w
	Abaxial hypodermis	-	+/--w	+/- w	+/--w						+ w	-	++ w	+ w				
	Adaxial parenchyma	+/--w, l	+/- w	++/- w	+/--w	+/--w	-	-	-		+/- w	-	++ w, p, c	+ w	+/- w	-	+ w	+/- w
	Abaxial parenchyma	+/--w, l	+/--w	+/- w	-						+ w	-	++ w	+ w				
	Xylem	-	-	-	-	-	-	-	-		-	-	-	-	-	-	-	-
**LM6**	Adaxial epidermis	-	-	-	-	++ w	+ w	++/- w	+ w	**LM20**	-	+/--w	+ w	+ w	-	-	+ w	+ w
	Abaxial epidermis	+/- w	++ w	+ w	-						+/- w	+/- w	+ w	+ w, l				
	Adaxial hypodermis	+ w	++ w	++ w	++ w	++ w	+ w	+ w, l	++ w, l		-	+/- w	+ w, l	+ w, l	-	-	+ w, l	++ w, l
	Abaxial hypodermis	+ w	++ w	++ w, l	+ w, l						+/--w	++/- w	+ w, l	+ w, l				
	Adaxial parenchyma	+/- w	++ w	++ w	+ w	+/- w	+/--w	+/--w	+/- w		+/--w	++/- w	+/- w	+ w	+/--w	+/--w	++/- w	++ w
	Abaxial parenchyma	++/- w	+ w	++ w	+ w						+/--w	++/- w	+ w	+ w				
	Xylem	-	-	-	+/- l	-	-	-	-		-	-	-	-	-	-	-	-

c – cytoplasm, l – middle lamella, p – plasma membrane, w – cell wall; ++ very strong signal, + strong signal, ++/- epitope present in the most of cells, +/- epitope present in the part of cells, +/--epitope present in the individual cells;—epitope not detected.

#### Pectic epitopes, leaf lamina, shady side (Table 7)

The LM5 antibody was present in both of the analyzed variants, but the signal intensity was stronger in injured leaves. The LM6 epitope was differentially distributed in the epidermis, which was manifested by its presence only in injured leaves (Fig 7E and 7F). The LM19 signal was very strong in all of the analyzed samples; however, the only difference was the presence of LM19 in the cytoplasm of injured leaves (Fig 8C and 8D). There were no significant differences in the occurrence of the LM20 epitope.

The distribution of pectic epitopes in all of the analyzed leaf lamina samples revealed statistically significant differences between healthy and injured leaves for the LM6, LM19 and LM20 epitopes.

#### Pectic epitopes, petiole, sunlit side (Table 7)

In healthy leaves, the LM5 epitope was present in the epidermal, hypodermal and individual parenchyma cell walls, whereas in injured leaves, LM5 was present only in the epidermal cell walls and the cytoplasm (Fig 6E and 6F). The LM19 epitope was detected in most of the epidermal, hypodermal and parenchyma cell walls only in healthy leaves. There were no significant differences in the occurrence of the LM20 epitope in any of the analyzed samples. Additionally, there were only slight differences in the occurrence of the LM6 epitope in the analyzed petioles, where the fluorescence of this epitope was more intensive in healthy leaves.

#### Pectic epitopes, petiole, shady side (Table 7)

The LM5 epitope occurred in the hypodermal cells and was more abundant in healthy leaves, whereas a weak LM5 signal in the epidermal walls was detected only in injured leaves. In injured leaves, the LM19 epitope was more abundant in the epidermis, hypodermis and parenchyma than in healthy leaves, whereas the LM19 signal was weaker and was not observed in all of the cells. The LM20 epitope in both samples was present in the epidermal and parenchyma walls and in the cell walls and middle lamella of the hypodermis; however, in injured leaves, LM20 was detected in the walls that surrounded the intracellular spaces. There were no significant differences in the occurrence of the LM6 epitope in either of the analyzed variants.

The distribution of pectic epitopes in all of the analyzed petioles revealed statistically significant differences between healthy and injured leaves for the LM5, LM19 and LM20 epitopes, including the level of light (Table 2).

## Discussion

In response to salinity, plants, and trees in particular, have developed a variety of defense mechanisms that allow them to minimize the effects of stress and maintain homeostasis [57–59]. The harmful influence of salinity on the growth and development of trees is well known and includes, among others, a reduction in growth, changes in hormone concentrations [60], vessel numbers and diameters [61,62], and cell wall thicknesses, as well as modification of the wood chemical composition [63].

The results presented here show that trees growing in the same salinity stress indicate a correlation between changes in the chemical composition of the cell wall and the content of prenyl lipids, and the health status of trees. Analysis of the correlation between the prenols and the chemical components of the cell wall showed a positive correlation between JIM13 in the leaf lamina and petiole and the LM6 antibody in the petiole. A negative correlation was observed between JIM16 and JIM8 in the case of the petiole and LM6, LM5 and LM20 in the case of the leaf lamina. These results suggest that some trees have developed resistance mechanisms in which changed levels of prenols and changes in chemical composition of the cell wall may be involved.

### Chemistry

The normal level of chloride in linden leaves should be less than 0.3%, but the threshold value beyond which visible leaf damage appears has been estimated as 0.6–0.8% [64–67]; thus, the first faint signs of leaf damage appear at 0.37% of the value of the chloride concentration [45]. This level was exceeded in the leaves of all of the studied variants.

The content of sodium also had a significant impact on the condition of the leaves. In injured leaves, the sodium level was five times higher than in healthy leaves. Sodium has a high concentration in soil and plants, and its excess mostly results in an ionic imbalance rather than a direct toxic effect [67–69].

The prenyl lipid content in the leaves decreased along with deterioration of the condition of the trees [30]. Skorupińska-Tudek et al. [53] found an increase in dolichols in the roots of *Coluria geoides* and *Cucumis sativus* in response to salinity, heavy metals, low temperatures and a lack of potassium and phosphorus in the nutrient medium. Furthermore, prenyl lipids may have a protective effect in the case of a viral infection in plants [29,70]. A negative correlation between the sodium and chloride content and that of polyprenols was observed in this study, which may suggest their protective role against adverse environmental conditions. Furthermore, the decline in the polyprenol concentration that was noted at higher levels of chloride and sodium likely indicates an enhancement of their catabolism under such conditions. This observation is in line with the supposition that poly-isoprenoids, which are scavengers of the reactive oxygen species that are massively generated upon stress, undergo decomposition [29]. Modulation of the content of poly-isoprenoids in response to chlorine and sodium salt concentration are in line with the postulated mechanism according to which poly-isoprenoids protect the biological membranes by ‘shielding’ other lipids and integral membrane proteins [29]. Such a role for volatile isoprenoids, i.e. their cooperation with carotene and tocopherols in order to fortify the defense system upon oxidative stress [71] has also been postulated. The ROS-scavenging function of poly-isoprenoid lipids, which are components of the membranes, seems to be of special importance. Poly-isoprenoids might be dedicated to scavenge the ROS that are generated by integral membrane enzymes, e.g. the plasma membrane NADPH oxidase. In this context, it seems plausible to assume, that membrane lipids might serve as the first targets for the destructive ROS species since it is energetically cheaper to remove and replace a damaged lipid molecule than a protein molecule. In line with this concept, the susceptibility of poly-isoprenoids to ROS (singlet oxygen, hydrogen peroxide etc.) has recently been documented [72].

Moreover, the present study showed a significant effect of light on the prenyl lipid content in the leaves of Crimean linden. Leaves growing in the sunlight contained more lipid prenyl than leaves growing in shade. Similarly, a significant effect of light on the prenyl lipid content was shown by Bajda et al. [36] in studies conducted on gymnosperm and angiosperm plants. It is postulated that tissues that are exposed to direct sunlit accumulate higher amounts of polyprenols [36].

### AGPs and pectins

AGPs have been implicated in many processes that are involved in plant growth and development [25,73–78]. Very few studies on changes in the cell wall components in trees growing under salt stress have been conducted recently [17,63]. In general, it is known that salinity affects the AGP levels [24], but detailed information concerning the presence of particular AGP epitopes in the cell wall under salt stress is not known [79]. The only data available in the literature (at least to the best of our knowledge), in which similar AGP antibodies were used, were from studies on the tracheid differentiation of *Pinus radiata* [80].

From among the wall proteins, arabinogalactan proteins (AGPs) are considered to be the most reactive in the response of cells to factors reaching the cell from the inside and outside. Because of the widespread occurrence of AGPs in plants and their conservative nature, it is postulated that they play an important role in the control of the growth and differentiation of plants [18–22]. At present, it is known that AGPs participate in the exchange of information between plant cells under different developmental condition [81–83].

Our results show spatial differences in the occurrence of the AGP epitope between healthy and injured trees and that for most antibodies, the differences were statistically significant. In general, the AGPs of the leaf lamina in healthy trees were more abundant than in injured leaves, and the greatest difference concerned the JIM16 antibodies, which were not present in injured leaves. Studies on a BY-2 tobacco culture showed that salt stress upregulated the total AGPs in the salt-adapted BY-2 cells [24]. Moreover, analysis of the gene expression in rice plants showed a strong up-regulation of AGP genes under salt stress [84]. Our results and the small amount of data from the literature suggest that increasing levels of AGPs in healthy leaves can be considered to be an adaptation to salinity. Studies on the effect of temperature stress on banana showed an abundance of the JIM16 epitope in the tolerant cultivar [85]. Based on these findings two general roles for classical AGPs were proposed: 1) they may stabilize plasma membranes subjected to high internal hydrostatic pressures by acting as periplasmic cushion or 2) AGPs *in muro* as pectic plasticizers [24]. The increase of AGPs outside a cell may be related to the stress response and stress adaptation of a plant to salinity as was shown on the example of a suspension culture of *Dactylis glomerata* [26]. It is postulate that the release of different AGPs in response to different salt concentration suggests the involvement of AGPs in multiple processes that are related to stress adaptation and cell-to-cell signaling [26]. Studies on *Medicago sativa* showed an increase of the AGPs that are recognized by the JIM8 antibody in NaCl-treated plants [27].

The abundance of AGPs on cell surfaces, and the presence of GPI anchors indicate that they are involved in cell-to-cell signaling [80]. AGPs have also been found within the cell walls [86–88]. Moreover, AGPs have been described as having adhesive properties and ability to association with other macromolecules [89]. This supposition led to the suggestion that their presence in the middle lamella and cell wall could serve as an adhesive for cell-to-cell contacts [80].

In the case of *Medicago sativa*, it was shown that there is an increase in the AGPs that are recognized by the JIM8 antibody in NaCl-treated plants [27]. This antibody showed no differences between healthy and injured trees in our studies and further analysis should be performed in the future to explain such a result.

Pectins are subjected to a number of modifications within the cell wall [90]. Changes in the composition of pectins have been described for different plants in various developmental stages and under the influence of biotic and abiotic factors [24,56,91–93]. In aspen hybrids grown under salt stress, it was shown that the presence of the pectic epitopes that are recognized by the JIM7 antibodies increased in comparison to the control plants, although the presence of the JIM5 antibodies was the same in both the salt-treated and non-treated plants [17]. Studies on *Medicago sativa* showed an increase of unesterified homogalacturonans (JIM5) under salt stress [27]. The lack of low-estrified pectins that are detected by the LM19 antibody in injured leaves in *T*. *x euchlora* was similar to the results for the aspen hybrid and may suggest that the rigidity of the cell walls was diminished in comparison to healthy leaves. The level of the LM20 pectic epitope was influenced by the health status of the trees and the level of light. In light of these results, at present, it is difficult to provide an unambiguous determination of the role of this pectic epitope in the physico-chemical properties of the wall and further studies are needed.

It is postulated that RG-I arabinans (LM6) are required for the maintenance of cell wall flexibility and that RG-I galactans (LM5) may be associated with the stiffening of the wall and firmness of the cell [92,94–96]. In *T*. *x euchlora* leaves, the LM5 epitope was abundantly present in all of the analyzed tissues, and no statistically significant differences were found between healthy and injured leaves. In *T*. *x euchlora*, a higher number of LM6 antibodies was detected in injured trees, and this difference was statistically significant. The increase in LM6 antibodies was described as a reaction of the plant to infection [8], which may suggest that this epitope is involved in the reaction of plants to different stressors.

## Conclusions

For the first time, the detailed spatial distribution of AGP (JIM8, JIM16, JIM13, and LM2) and pectic (LM5, LM6, LM19, and LM20) epitopes in different tissues of *T*. *x euchlora* leaves was determined both in the leaf lamina and the petiole from trees grown under the same level of salt stress but exhibiting differences in their health status. Our results extend the knowledge about the presence of pectic and AGP epitopes in connection with salt stress on the example of a deciduous tree species. It can be postulated that the substitution of one chemical pattern of cell wall composition for another may reflect a local remodeling of the cell wall.

The obtained results suggest that the defensive strategy of *T*. *x euchlora* trees to salt stress may rely on 1) increasing the synthesis of prenyl lipids that might act as scavengers of reactive oxygen species and/or modulate the transport and deposition of chloride and sodium in the leaf cells, and 2) changing the chemical composition of pectin and AGPs in the cell walls.

Our results can be connected with the health status of trees, which suggests that the adaptation of trees to salinity can be characterized by different chemical compositions in the cell walls.

## References

[1] Fricke W. Rapid and tissue-specific accumulation of solutes in the growth zone of barley leaves in response to salinity. Planta. 2004;219: 515–525. 10.1007/s00425-004-1263-0 15085434

[2] Fricke W, Akhiyarova G, Wei W, Alexandersson E, Miller A, Kjellbom PO, et al The short-term growth response to salt of the developing barley leaf. J Exp Bot. 2006;57: 1079–1095. 10.1093/jxb/erj095 16513814

[3] Munns R, Tester M. Mechanisms of salinity tolerance. Annu Rev Plant Physiol. 2008;59: 651–681. 10.1146/annurev.arplant.59.032607.092911 18444910

[4] Al-Abdoulhadi IA. Influence of salinity stress on photosynthesis and chlorophyll content in date palm (*Phoenix dactylifera* L.) cultivars. Afr J Agric Res. 2012;7: 3314–3319.

[5] Dmuchowski W, Baczewska AH, Gozdowski D, Brągoszewska P. Effect of salt stress on the chemical composition of leaves of different trees species in urban environment. Fresen Environ Bull. 2013;22: 987–994.

[6] Verhertbruggen Y, Knox JP. Pectic polysaccharides and expanding cell walls In: Verbelen JP, Vissenberg K (eds) Plant cell monographs 5. Springer: Berlin, Expanding Cell, 2006; pp 139–158.

[7] Palin R, Geitmann A. The role of pectin in plant morphogenesis. BioSystems. 2012;109: 397–402. 10.1016/j.biosystems.2012.04.006 22554809

[8] Johnsen HR, Striberny B, Olsen S, Vidal-Melgosa S, Fangel JU, Willats WGT, et al Cell wall composition profiling of parasitic giant dodder (*Cuscuta reflexa*) and its hosts: a priori differences and induced changes. New Phytol. 2015;207: 805–816. 10.1111/nph.13378 25808919

[9] Schmohl N, Horst WJ. Cell wall pectin content modulates aluminum sensitivity of *Zea mays* (L.) cells grown in suspension culture. Plant Cell Environ. 2000;23: 735–742.

[10] Li Y, Sperry JS, Shao M. Hydraulic conductance and vulnerability to cavitation in corn (*Zea mays* L.) hybrids of differing drought resistance. Environ Exp Bot. 2009;66: 341–346.

[11] Fernandes JC, García-Angulo P, Goulao LF, Acebes JL, Amâncio S. Mineral stress affects the cell wall composition of grapevine (*Vitis vinifera* L.) callus. Plant Sci. 2013;205–206: 111–120. 10.1016/j.plantsci.2013.01.013 23498868

[12] Lima RB, Dos Santos TB, Vieira LG, Ferrarese ML, Ferrarese-Filho O, Donatti L, et al Heat stress causes alterations in the cell-wall polymers and anatomy of coffee leaves (*Coffea arabica* L.). Carbohydr Polym. 2013;93: 135–143. 10.1016/j.carbpol.2012.05.015 23465912

[13] Baldwin L, Domon JM, Klimek JF, Fournet F, Sellier H, Gillet F, et al Structural alteration of cell wall pectins accompanies pea development in response to cold. Phytochem. 2014;104: 37–47. 10.1016/j.phytochem.2014.04.011 24837358

[14] Kreuzwieser J, Rennenberg H. Molecular and physiological responses of trees to waterlogging stress. Plant Cell Environ. 2014;37: 2245–2259. 10.1111/pce.12310 24611781

[15] Sasidharan R, Voesenek LACJ, Pierik R. Cell-wall modifying proteins mediate plant acclimatization to biotic and abiotic stresses. Crit Rev Plant Sci. 2011;30: 548–562.

[16] Iraki NM, Bressan RA, Carpita NC. Extracellular polysaccharides and proteins of tobacco cell cultures and changes in composition associated with growth-limiting adaptation to water and saline stress. Plant Physiol. 1989;91: 54–61. 16667043 10.1104/pp.91.1.54 PMC1061951

[17] Muszynska A, Jarocka K, Kurczynska EU. Plasma membrane and cell wall properties of an aspen hybrid (*Populus tremula x tremuloides*) parenchyma cells under the influence of salt stress. Acta Physiol Plant. 2014;36: 1155–1165.

[18] Samson MR, Jongeneel R, Klis FM. Arabinogalactan protein in the extracellular space of *Phaseolus vulgaris* hypocotyls. Phytochem. 1984;23: 493–496.

[19] Basile DV, Basile MR. The occurrence of cell wall-associated arabinogalactan proteins in the Hepaticae. Bryologist. 1987;90: 401–404.

[20] Pennell RI, Knox JP, Scofield GN, Selvendran RR, Roberts K. Related to the arabinogalactan proteins is unique to flowering plants. J Cell Biol. 1989;108: 1967–1977. 2469683 10.1083/jcb.108.5.1967 PMC2115552

[21] Komalavilas P, Zhu JK, Nothnagel EA. Arabinogalactan-proteins from the suspension culture medium and plasma membrane of rose cells. J Biol Chem. 1991;266: 15956–15965. 1874742

[22] Nothnagel EA. Proteoglycans and related components in plant cells. Int Rev Cytol. 1997;174: 195–291. 9161008 10.1016/s0074-7696(08)62118-x

[23] Li SX, Showalter AM. Cloning and developmental/stress-regulated expression of a gene encoding a tomato arabinogalactan protein. Plant Mol Biol. 1996;32: 641–652. 8980516 10.1007/BF00020205

[24] Lamport DTA, Kieliszewski MJ, Allan M. Showalter AM. Salt stress upregulates periplasmic arabinogalactan proteins: using salt stress to analyse AGP function. New Phytol. 2006;169: 479–492. 10.1111/j.1469-8137.2005.01591.x 16411951

[25] Ouyang B, Yang T, Li H, Zhang L, Zhang Y, Zhang J, et al Identification of early salt stress response genes in tomato root by suppression subtractive hybridization and microarray analysis. J Exp Bot. 2006;58: 507–520. 10.1093/jxb/erl258 17210988

[26] Zagorchev L, Petrova S, Odjakova M. Arabinogalactan proteins in salt adapted suspension cultures of *Dactylis glomerata*. Gen Appl Plant Physiol. 2008;34: 159–168.

[27] Boughanmi N, Thibault F, Decou R, Fleurat-Lessard P, Béré E, Costa G, et al NaCl effect on the distribution of wall ingrowth polymers and arabinogalactan proteins in type A transfer cells of *Medicago sativa* Gabèsleaves. Protoplasma. 2010;242: 69–80. 10.1007/s00709-010-0125-9 20237812

[28] Heidarvand L, Amiri RM. What happens in plant molecular responses to cold stress? Acta Physiol Plant. 2010;32: 419–431.

[29] Bajda A, Konopka-Postupolska D, Krzymowska M, Hennig J, Skorupinska-Tudek K, Surmacz L, et al Role of polyisoprenoids in tobacco resistance against biotic stresses. Physiol Plant. 2009;135: 351–364. 10.1111/j.1399-3054.2009.01204.x 19292825

[30] Baczewska AH, Dmuchowski W, Jozwiak A, Gozdowski D, Brągoszewska P, Dąbrowski P. Effect of salt stress on prenol lipids in the leaves of *Tilia* ‘Euchlora’. Dendrobiology. 2014;72: 177–186.

[31] Gutkowska M, Bienkowski T, Hung VS, Wanke M, Hertel J, Danikiewicz W, et al Proteins are polyisoprenylated in *Arabidopsis thaliana*. Biochem Biophys Res Commun. 2004;322: 998–1004. 10.1016/j.bbrc.2004.08.025 15336563

[32] Hjertman M, Wejde J, Dricu A, Carlberg M, Griffiths WJ, Sjovall J, et al Evidence for protein dolichylation. FEBS Lett. 1997;195: 755–761. 10.1016/s0014-5793(97)01208-8 9373159

[33] Skorupinska-Tudek K, Wojcik J, Swiezewska E. Polyisoprenoid alcohols – recent results of structural studies. Chem Rec. 2008;8: 33–45. 10.1002/tcr.20137 18302278

[34] Loreto F, Velikova V. Isoprene produced by leaves protects the photosynthetic apparatus against ozone damage, quenches ozone products, and reduces lipid peroxidation of cellular membranes. Plant Physiol. 2001;127: 1781–1787. 11743121 PMC133581

[35] Bergamini E. Dolichol: an essential part in the antioxidant machinery of cell membranes. Biogerontology. 2003;4: 337–339. 14756125 10.1023/b:bgen.0000006637.48753.07

[36] Bajda A, Chojnacki T, Hertel J, Swiezewska E, Wójcik J, Kaczkowska A, et al Light conditions alter accumulation of long chain polyprenols in leaves of trees and shrubs throughout the vegetation season. Acta Biochim Pol. 2005;52: 233–241. 15827620

[37] Sehmer L, Alaoui-sosse B, Dizengremel P. Effect of salt stress on growth and on the detoxifying pathway of pedunculate oak seedlings (*Quercus robur* L.). J Plant Physiol. 1995;147: 144–151.

[38] Czerniawska-Kusza I, Kusza G, Dużyński M. Effect of deicing salts on urban soils and health status of roadside trees in the Opole Region. Environ Toxicol. 2004;19: 296–301. 10.1002/tox.20037 15269899

[39] Franklin JA, Zwiazek JJ. Ion uptake in *Pinus banksiana* treated with sodium chloride and sodium sulphate. Physiol Plant. 2004; 120:482–490. 10.1111/j.0031-9317.2004.00246.x 15032846

[40] Oleksyn J, Kloeppel BD, Łukasiewicz S, Karolewski P, Reich PB. Ecophysiology of horse chestnut (*Aesculus hippocastanum* L.) in degraded and restored urban sites. Pol J Ecol. 2007;55: 245–260.

[41] Cekstere G, Nikodemus O, Osvalde A. Toxic impact of the de-icing material to street greenery in Riga, Latvia. Urban For Urban Green. 2008;7: 207–217.

[42] Green SM, Machin R, Cresser MS. Effect of long-term changes in soil chemistry induced by road salt applications on N-transformations in roadside soils. Environ Pollut. 2008;152: 20–31. 10.1016/j.envpol.2007.06.005 17640786

[43] Lax S, Peterson EW. Characterization of chloride transport in the unsaturated zone near salted road. Environ Geol. 2009;58: 1041–1049.

[44] Fay L, Shi X. Environmental impacts of chemicals for snow and ice control: State of the knowledge. Water Air Soil Pollut. 2012;223: 2751–2770.

[45] Dmuchowski W, Gozdowski D, Baczewska AH, Rutkowska B, Szulc W, Suwara I, et al Effect of salt stress caused by deicing on the content of microelements in the leaves of linden. J Elem. 2014;19: 65–79.

[46] Li C-H, Wang G, Zhao J-L, Zhang L-Q, Ai L-F, Han Y-F, et al The receptor-like kinase SIT1 mediates salt sensitivity by activating MAPK3/6 and regulating ethylene homeostasis in rice. Plant Cell. 2014;26: 2538–2553. 10.1105/tpc.114.125187 24907341 PMC4114950

[47] Dmuchowski W, Baczewska AH, Brągoszewska P. Reaction of street trees to adverse environmental conditions in the centre of Warsaw. EQ. 2011;15: 97–105.

[48] Liesebach H, Sinkó Z. A contribution to the signaling of the genus *Tilia* with respect to some hybrids by RAPD analysis. Dendrobiology. 2008;59: 13–22.

[49] Baczewska AH, Dmuchowski W, Gozdowski D, Brągoszewska P. Changes in health status and chemical composition of tree leaves of the Crimean linden in the years 2000 and 2009. Environ Protect Nat Resour. 2011;49: 84–95.

[50] Allen SE, Grimshaw HN, Parkinson JA, Quarmby C (eds). Chemical Analysis of Ecological Materials. 1974 Blackwell Scientific Publications, Oxford, UK.

[51] Perkin-Elmer (ed). Analytical methods for atomic absorption spectrophotometry 1990 Bodenseewerk, Prklin Elmer &Co., Ueberlingen.

[52] LaCroix RL, Keeney DR, Walsh LM. Potentiometric titration of chloride in plant tissue extracts using the chloride ion electrode. Commun Soil Sci Plant Anal. 1970;1: 1–6.

[53] Skorupinska-Tudek K, Bienkowski T, Olszowska O, Furmanowa M, Chojnacki T, Danikiewicz W, et al Divergent pattern of polyisoprenoid alcohols in the tissues of *Coluria geoides*: a new electrospray ionization MS approach. Lipids. 2003;38: 981–990. 14584606 10.1007/s11745-003-1152-3

[54] Jozwiak A, Pies M, Skorupińska-Tudek K, Kania M, Dydak M, Danikiewicz W, et al Sugar availability modulates polyisoprenoid and phytosterol profiles in *Arabidopsis thaliana* hairy root culture. Bba-Mol Cell Biol L. 2013;1831: 438–447. 10.1016/j.bbalip.2012.11.006 23178167

[55] Potocka I, Baldwin TC, Kurczynska EU. Distribution of lipid transfer protein 1 (LTP1) epitopes associated with morphogenic events during somatic embryogenesis of *Arabidopsis thaliana*. Plant Cell Rep. 2012;31: 2031–2045. 10.1007/s00299-012-1314-0 22821363 PMC3472069

[56] Sala K, Potocka I, Kurczynska E. Spatio-temporal distribution and methyl-esterification of pectic epitopes provide evidence of developmental regulation of pectins during somatic embryogenesis in *Arabidopsis thaliana*. Biol Plantarum. 2013;57: 410–416.

[57] Munns R. Comparative physiology of salt and water stress. Plant Cell Environ. 2002;25: 239–250. 11841667 10.1046/j.0016-8025.2001.00808.x

[58] Dajic Z. Physiology and Molecular Biology of Stress Tolerance in Plants In Madhava Rao KV, Raghavendra AS, Janardhan Reddy K, editors. Physiology and molecular biology of stress tolerance in plant. Dordrecht: Springer – Verlag; 2006 Pp. 41–99.

[59] Goździcka-Józefiak A, Woźny A. Reakcje komórek roślin na czynniki stresowe. Vol II. Poznań: Wydawnictwo Naukowe Uniwersytetu im. Adama Mickiewicza; 2010.

[60] Kozlowski TT. Responses of woody plants to flooding and salinity. Tree. 1997; 2010 17: 490–490.

[61] Sperry JS, Meinzer FC, McCulloh KA. Safety and efficiency conflicts in hydraulic architecture: scaling from tissues to trees. Plant Cell Environ. 2008;31: 632–645. 10.1111/j.1365-3040.2007.01765.x 18088335

[62] Teichmann T, Bolu-Arianto WH, Olbrich A, Langenfeld-Heyser R, Gobel C, Grzeganek P, et al GH3::GUS reflects cell specific developmental patterns and stress-induced changes in wood anatomy in the poplar stem. Tree Physiol. 2008;28: 1305–1315. 18595842 10.1093/treephys/28.9.1305

[63] Janz D, Lautner S, Wildhagen W, Behnke K, Schnitzler J-P, Rennenberg H, et al Salt stress induces the formation of a novel type of ‘pressure wood’ in two *Populus* species. New Phytol. 2012;94: 129–141. 10.1111/j.1469-8137.2011.03975.x 22126133

[64] Shortle WC, Rich AE. Relative sodium tolerance of common roadside trees in southeastern New Hampshire. Plant Dis Rep. 1970;54: 360–362.

[65] Pauleit S. Vitalitätskartierung von Stadtbäume in München. 1988 Garten Landschraft 7:38–40.

[66] Munck IA, Bennett CM, Camilli KS, Nowak RS. Long-term impact of de-icing salts on tree health in the Lake Tahoe Basin: Environmental influences and interactions with insects and diseases. Forest Ecol Manag. 2010;260: 1218–1229.

[67] Dmuchowski W, Brogowski Z, Baczewska AH. Evaluation of vigour and health of street trees in Warsaw using the foliar ionic status. Pol J Environ Stud. 2011;20: 489–496.

[68] Alaoui-Sosse B, Sehmer L, Barnola P, Diezengremel P. Effect of NaCl salinity on growth and mineral partitioning in *Quercus robur* L., a rhythmically growing species. Trees. 1998;12: 424–430.

[69] Kayama M, Quoreshi AM, Kitaoka S, Kitahashi Y, Sakamoto Y, Maruyama Y, et al Effects of deicing salt on the vitality and health of two spruce species, Piceaabies Karst., and Piceaglehnii Masters planted along roadsides in northern Japan. Environ Pollut. 2003;124: 127–137. 12683989 10.1016/s0269-7491(02)00415-3

[70] Surmacz L, Swiezewska E. Polyisoprenoids – secondary metabolites or physiologically important superlipids? Biochem Biophys Res Commun. 2011;407: 627–632. 10.1016/j.bbrc.2011.03.059 21419101

[71] Penuelas J, Munne-Bosch S. Isoprenoids: an evolutionary pool for photoprotection. Trends Plant Sci. 2005;10: 166–169. 10.1016/j.tplants.2005.02.005 15817417

[72] Komaszylo Née Siedlecka J, Kania M, Masnyk M, Cmoch P, Lozinska I, Czarnocki Z, et al Isoprenoid Alcohols are Susceptible to Oxidation with Singlet Oxygen and Hydroxyl Radicals. Lipids. 2016;51: 229–244. 10.1007/s11745-015-4104-y 26715533 PMC4735226

[73] Lind JL, Bönig I, Clarke AE, Anderson MA. A style-specific 120-kD glycoprotein enters pollen tubes of Nicotiana alata in vivo. Sex Plant Reprod. 1996; 9:75–96.

[74] Gao M, Showalter AM. Yariv reagent treatment induces programmed cell death in Arabidopsis cell cultures and implicates arabinogalactan protein involvement. Plant J. 1999;19: 321–331. 10476079 10.1046/j.1365-313x.1999.00544.x

[75] Van Hengel AJ, Van Kammen A, De Vries SC. A relationship between seed development, arabinogalactan-proteins (AGPs) and the AGP mediated promotion of somatic embryogenesis. Physiol Plant. 2002;114: 637–644. 11975739 10.1034/j.1399-3054.2002.1140418.x

[76] Van Hengel AJ, Roberts K. AtAGP30, an arabinogalactan-protein in the cell walls of the primary root, plays a role in root regeneration and seed germination. Plant J. 2003;36: 256–270. 14535889 10.1046/j.1365-313x.2003.01874.x

[77] Park MH, Suzuki Y, Chono M, Knox JP, Yamaguchi I. CsAGP1, a signaling ns-responsive gene from cucumber hypocotyls, encodes a classical arabinogalactan protein and is involved in stem elongation. Plant Physiol. 2003;131: 1450–1459. 10.1104/pp.015628 12644694 PMC166904

[78] Ellis M, Egelund J, Schultz CJ, Bacic A. Arabinogalactan-Proteins: Key Regulators at the Cell Surface? Plant Physiol. 2010;153: 403–419. 10.1104/pp.110.156000 20388666 PMC2879789

[79] Gall H, Philippe F, Domon J-M, Gillet F, Pelloux J, Rayon C. Cell Wall Metabolism in Response to Abiotic Stress. Plants. 2015;4: 112–166. 10.3390/plants4010112 27135320 PMC4844334

[80] Putoczki TL, Pettolino F, Griffin MDW, Möller R, Gerrard JA, Bacic A, et al Characterization of the structure, expression and function of *Pinus radiata* D. Don arabinogalactan-proteins. Planta. 2007;226: 1131–1142. 10.1007/s00425-007-0559-2 17569081

[81] Youl JJ, Bacic A, Oxley D. Arabinogalactan-proteins from *Nicotiana alata* and *Pyrus communis* contain glycosylphosphatidylinositol membrane anchors. Proc Natl Acad Sci U S A. 1998;95: 7921–7926. 9653116 10.1073/pnas.95.14.7921 PMC20905

[82] Schultz CJ, Johnson KL, Currie G, Bacic A. The classical arabinogalactan protein gene family of Arabidopsis. Plant Cell. 2000;12: 1751–1768. 11006345 10.1105/tpc.12.9.1751 PMC149083

[83] Svetek J, Yadav MP, Nothnagel EA. Presence of a glycosylphosphatidylinositol lipid anchor on rose arabinogalactan proteins. J Biol Chem. 1999;274: 14724–14733. 10329668 10.1074/jbc.274.21.14724

[84] Ma H, Zhao J. Genome-wide identification, classification, and expression analysis of the arabinogalactan protein gene family in rice (*Oryza sativa* L.). J Exp Bot. 2010;61: 2647–2668. 10.1093/jxb/erq104 20423940 PMC2882264

[85] Yan Y, Takac T, Li X, Chen H, Wang Y, Xu E, et al Variable content and distribution of arabinogalactan proteins in banana (Musa spp.) under low temperature stress. Front Plant Sci. 2015;6: 1–14. 26074928 10.3389/fpls.2015.00353 PMC4444754

[86] Schopfer P. Cytochemical identification of arabinogalactan protein in the outer epidermal wall of maize coleoptiles. Planta. 1990;183: 139–142. 10.1007/BF00197578 24193543

[87] Li Y-Q, Bruun L, Pierson ES, Cresti M. Periodic deposition of arabinogalactan epitopes in the cell wall of pollen tubes of *Nicotiana tabacum* L. Planta. 1992;188: 532–538. 10.1007/BF00197045 24178385

[88] Serpe MD, Nothnagel EA. Effects of Yariv phenylglycosides on Rosa cell suspensions: evidence for the involvement of arabi- nogalactan-proteins in cell proliferation. Planta. 1994;193: 542–550.

[89] Clarke AE, Anderson RL, Stone BA. Form and function of arabinogalactans and arabinogalactan-proteins. Phytochem. 1979;18: 521–540.

[90] Cosgrove DJ. Relaxation in a high-stress environment: the molecular bases of extensible cell walls and cell enlargement. Plant Cell. 1997;9: 1031–1041. 10.1105/tpc.9.7.1031 9254929 PMC156977

[91] Willats WG, Knox JP. Immunoprofiling of pectic polysaccharides. Anal Biochem. 1999;268: 143–146. 10.1006/abio.1998.3039 10036173

[92] Talboys PJ, Zhang HM, Paul Knox J. ABA signaling modulates the detection of the LM6 arabinan cell wall epitope at the surface of *Arabidopsis thaliana* seedling root apices. New Phytol. 2011;190: 618–626. 10.1111/j.1469-8137.2010.03625.x 21275992

[93] Munns R, Gilliham M. Salinity tolerance of crops – what is the cost? New Phytol. 2015;208: 668–673. 10.1111/nph.13519 26108441

[94] McCartney L, Ormerod AP, Gidley MJ, Knox JP. Temporal and spatial regulation of pectic (1->4)-beta-D-galactan in cell walls of developing pea cotyledons: implications for mechanical properties. Plant J. 2000;22: 105–113. 10792826 10.1046/j.1365-313x.2000.00719.x

[95] Jones L, Milne JL, Ashford D, McQueen-Mason SJ. Cell wall arabinan is essential for guard cell function. Proc Natl Acad Sci U S A. 2003;100: 11783–11788. 10.1073/pnas.1832434100 13130074 PMC208835

[96] Ulvskov P, Wium H, Bruce D, Jørgensen B, Qvist KB, Skjøt M, et al Biophysical consequences of remodeling the neutral side chains of rhamnogalacturonan I in tubers of transgenic potatoes. Planta. 2005;220: 609–620. 10.1007/s00425-004-1373-8 15517357

